# Outcomes and mechanical complications of acute myocardial infarction during the second wave pandemic in a Milan HUB center for cardiac emergencies

**DOI:** 10.3389/fcvm.2022.950952

**Published:** 2022-10-03

**Authors:** Marco Penso, Antonio Frappampina, Nicola Cosentino, Gloria Tamborini, Fabrizio Celeste, Monica Ianniruberto, Paolo Ravagnani, Sarah Troiano, Giancarlo Marenzi, Mauro Pepi

**Affiliations:** ^1^Centro Cardiologico Monzino IRCCS, Milan, Italy; ^2^Department of Electronics, Information and Biomedical Engineering, Politecnico di Milano, Milan, Italy

**Keywords:** STEMI, COVID-19, coronavirus disease, mechanical complications, acute myocardial infarction

## Abstract

**Aims:**

COVID-19 has dramatically impacted the healthcare system. Evidence from previous studies suggests a decline in in-hospital admissions for acute myocardial infarction (AMI) during the pandemic. However, the effect of the pandemic on mechanical complications (MC) in acute ST-segment elevation myocardial infarction (STEMI) has not been comprehensively investigated. Therefore, we evaluated the impact of the pandemic on MC and in-hospital outcomes in STEMI during the second wave, in which there was a huge SARS-CoV-2 diffusion in Italy.

**Methods and results:**

Based on a single center cohort of AMI patients admitted with STEMI between February 1, 2019, and February 28, 2021, we compared the characteristics and outcomes of STEMI patients treated during the pandemic vs. those treated before the pandemic. In total, 479 STEMI patients were included, of which 64.5% were during the pandemic. Relative to before the pandemic, primary percutaneous coronary intervention (PCI) declined (87.7 vs. 94.7%, *p* = 0.014) during the pandemic. Compared to those admitted before the pandemic (10/2019 to 2/2020), STEMI patients admitted during the second wave (10/2020 to 2/2021) presented with a symptom onset-to-door time greater than 24 h (26.1 vs. 10.3%, *p* = 0.009) and a reduction of primary PCI (85.2 vs. 97.1%, *p* = 0.009). MC occurred more often in patients admitted during the second wave of the pandemic than in those admitted before the pandemic (7.0 vs. 0.0%, *p* = 0.032). In-hospital mortality increased during the second wave (10.6 vs. 2.9%, *p* = 0.058).

**Conclusion:**

Although the experience gained during the first wave and a more advanced hub-and-spoke system for cardiovascular emergencies persists, late hospitalizations and a high incidence of mechanical complications in STEMI were observed even in the second wave.

## Introduction

The global outbreak of coronavirus disease 2019 (COVID-19) has led to not only a significant number of deaths and morbidities but has also impacted other non-COVID-19 conditions, including cardiovascular ones (1). Indeed, a growing amount of data suggests a dramatic decline in hospital admissions for acute myocardial infarction (AMI) worldwide during the COVID-19 pandemic, mostly because patients did not activate emergency medical systems because hospitals were perceived as dangerous places due to the infection risk (2–6). Moreover, several hospitals were dedicated to COVID-19 emergencies. Most intensive therapy beds, including those in intensive cardiac care units, have been dedicated to treating patients with pneumonia and severe acute respiratory syndrome. For this reason, the government of Lombardy (Italy), one of the first countries hit by the pandemic outside China, and local health authorities decided to centralize the treatment of cardiovascular emergencies in a limited number of centers. In particular, a big hub-and-spoke model for cardiovascular, either cardiological or cardiac surgical emergencies, was built up soon after the COVID-19 outbreak to converge treatment of acute coronary syndrome (ACS) in dedicated centers active 24/7 in the region, implementing availability of intensive care unit beds in general hospitals converted to COVID-19 treatment. Therefore, our cardiology institute (Centro Cardiologico Monzino, Milan) became one of the four referral centers for cardiovascular emergencies in this regional hub-and-spoke system, and we, therefore, admitted and treated an increased number of AMI patients, compared with the decline in hospitalizations for AMI observed overall in our region and other countries.

It is also well-known that, during the COVID-19 pandemic, patients with AMI had a significantly higher in-hospital mortality than those admitted before COVID-19, potentially due to their late arrival at the hospital. In this critical clinical setting, mechanical complications (MC) and cardiogenic shock are rare but disastrous complications with a poor prognosis (7). Data on MC rates during the COVID-19 pandemic are scarce. However, delays and lack of prompt pharmacologic or invasive reperfusion therapies during the COVID-19 pandemic might have been linked to increased AMI complications.

In this study, we aimed to compare in-hospital outcomes of AMI patients admitted before (October 2019–February 2020) and during the pandemic (March 2020–February 2021), with a special focus on the incidence of MC between the two study periods. Moreover, we believe that the second pandemic wave, mainly thanks to knowledge and experience gained during the first wave and a more advanced hub-and-spoke system, may be more representative of the impact of COVID-19 on acute cardiovascular disease; we compared the hospital outcomes, and MC rates of AMI patients hospitalized only during the second wave (October 2020–February 2021) with those admitted before the pandemic.

## Materials and methods

### Study population

**FIGURE 1:**
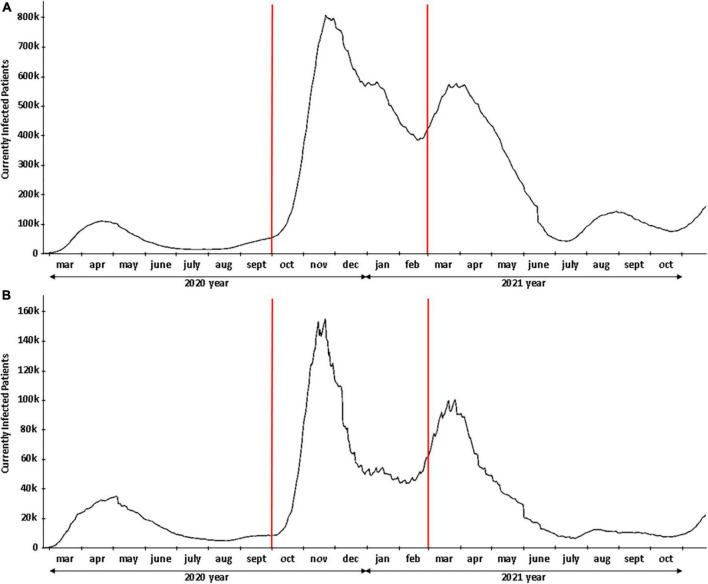
COVID-19 pandemic profile. **(A)** Cases in Italy. **(B)** Cases in the Lombardy region. The red lines delineate the second wave of COVID-19.

This retrospective study was carried out on a consecutive cohort of AMI patients admitted to Cardiac Center Monzino IRCCS (Milan, Italy) with acute ST-segment elevation myocardial infarction (STEMI) between February 1, 2019, and February 28, 2021. Patients underwent primary PCI if they had typical chest pain within 12 h (24 h for those with cardiogenic shock) and ST-segment elevation of at least 1 mm in two or more contiguous leads or a new left bundle branch block. The exclusion criteria were non-evidence of atherothrombotic coronary artery disease at angiography (e.g., vasospastic angina or coronary dissection). The history of each patient was recorded at the time of hospital admission in a single electronic database, and the identification of STEMI was based on data generated at hospital discharge. In order to compare the clinical characteristics, management, and outcomes of STEMI patients before and during the COVID-19 period, we decided to consider the cut-off date for the introduction of the state of sanitary emergency for the COVID-19 pandemic in Italy (March 1, 2020). Therefore, the study population was categorized into two periods: from March 2020 to February 2021 (during the pandemic) and from February 2019 to February 2020 (before the pandemic). To outline the characteristics of STEMI patients admitted during the second COVID-19 wave period (Figure 1), patients admitted for STEMI from October 2020 to February 2021 were identified and compared with those admitted before the COVID-19 pandemic (from October 2019 to February 2020). The study complied with the Declaration of Helsinki. The ethics committee approved the research, and informed consent was obtained from each subject.

### Clinical variables and definitions

**FIGURE 2:**
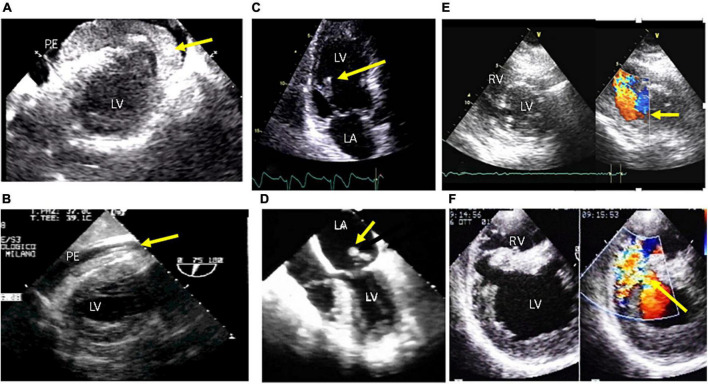
Echocardiographic aspects of mechanical complications of acute myocardial infarction. **Upper panel:** transthoracic echo. **Lower panel:** transesophageal echo. LV, left ventricle; RV, right ventricle. **(A,B)** Free wall rupture with pericardial effusion (PE) and thrombosis intrapericardial hematoma in the pericardial space (yellow arrows); **(C)** partial papillary muscle rupture (yellow arrow); **(D)** Complete papillary muscle rupture (yellow arrow) with eversion in the left atrium (LA); **(E,F)** ventricular septal defect with left-to-right shunt across the septum (yellow arrows).

Evaluation of patients included medical history, electrocardiography (ECG), blood examination, transthoracic echocardiography (TTE), and coronary angiography. In addition to demographic characteristics (age, sex, body mass index [BMI]), and cardiovascular risk factors (including arterial hypertension, current smoking, dyslipidemia, diabetes mellitus, prior cardiovascular events, etc.), the following variables were collected: medical therapy before admission and during hospitalization; Killip class; ischemic time (time from symptoms’ onset to hospital admission); Thrombolysis In Myocardial Infarction (TIMI) flow grade before and after PCI; left ventricular (LV) ejection fraction (LVEF); intensive coronary care unit length of stay; MC; concomitant SARS-CoV-19 infection. SARS-CoV-19 infection was defined by molecular testing on nasopharyngeal swabs or bronchoalveolar lavage fluid at presentation. The decision to perform PCI or surgical approach for MC was decided by the heart team according to guidelines (8, 9). MCs included: rupture of the LV free wall defined as an abrupt tear in the infarcted myocardium causing hemopericardium and cardiac tamponade; rupture of the papillary muscle defined as a tear in the infarcted mitral subvalvular apparatus causing acute mitral regurgitation; and ventricular septal rupture defined as a tear in the infarcted interventricular septum with evidence of a shunt between the left and right ventricle (Figure 2). TTE was performed in all patients soon after hospital admission and then repeated during hospitalization, using commercially available ultrasound systems (Vivid E9 and E95; GE Medical Systems, Horten, Norway; and iE33 and Epiq; Philips Medical Systems, Andover, Massachusetts) in the parasternal (long- and short-axis) and apical (2-, 3-, and 4-chamber) views. Echocardiographic measurements were made in accordance with guidelines (10). Transesophageal echocardiography (TEE) was also performed when TTE was suboptimal to confirm and complete TTE evaluation, particularly in ventilated patients and all cases undergoing surgery.

### Statistical analysis

Statistical analysis was performed with SPSS, version 27 software (SPSS, Inc., Chicago, IL, USA). Continuous variables were expressed as mean ± standard deviation (SD) or median (25th–75th percentile) as appropriate, and discrete variables as absolute numbers and percentages. Student-independent *t*- or Mann–Whitney *U* tests were used as appropriate to compare continuous variables between patients during and before the pandemic. Comparisons between groups of discrete variables were performed by χ^2^ or Fisher exact test if the expected cell count was < 5. Logistic regression analysis was used to assess independent predictors of MCs (results presented as odds ratio and 95% confidence interval). Variables with a *P* < 0.05 in univariate analysis were included in a multivariate logistic regression analysis with a stepwise selection procedure for identifying independent variables predicting MCs. All tests were two-tailed, and a *P*-value < 0.05 was considered statistically significant.

## Results

A total of 479 STEMI patients were included in the analysis from February 2019 to February 2021. Of them, 170 (35.5%) were admitted before the pandemic, whereas 309 (64.5%) were admitted during the pandemic. The study population comprised 367 men and 112 women (mean age, 67 ± 12 years). The number of STEMI patients positive for SARS-CoV-2 infection was 7 (2.3%). Patients’ characteristics before and during the COVID-19 period are listed in Table 1. Cardiovascular risk factors (arterial hypertension, diabetes mellitus, dyslipidemia), LVEF at presentation, and arrhythmic complications (atrial fibrillation and/or ventricular arrhythmias) were similar in the study groups, regardless of the temporal window considered. Patients undergoing primary PCI within 24 h of the onset of symptoms had significantly fewer symptoms during the pandemic than those before the pandemic (87.7 vs. 94.7%, *p* = 0.014). During the pandemic, a higher incidence of intraventricular thrombosis (6.1 vs. 1.2%, *p* = 0.01) was observed compared to the previous no-pandemic year. There was no significant difference in MC rate between the two study periods, despite a clear trend toward more MC in the pandemic period (13 cases vs. 3 cases, respectively). Furthermore, in-hospital deaths (18.1 vs. 14.7%, *p* = 0.218) were higher during the pandemic, but the difference was insignificant.

**TABLE 1:** Baseline and clinical characteristics of the study population before and during the pandemic.

	All patient (*n* = 479)	Before pandemic (*n* = 170)	During pandemic (*n* = 309)	*P*-value
**Demographic characteristics**				
Age, years	67 ± 12	66 ± 12	67 ± 12	0.302
Female	112 (23.4%)	37 (21.8%)	75 (24.3%)	0.535
Body mass index, kg/m^2^	26.6 ± 4.4	27.0 ± 5.0	26.4 ± 4.0	0.170
**Cardiovascular risk factors**				
Hypertension	273 (57.0%)	98 (57.6%)	175 (56.6%)	0.830
Current smoking	138 (28.8%)	86 (50.6%)	172 (55.7%)	0.440
Dyslipidaemia	188 (39.2%)	75 (44.1%)	113 (36.6%)	0.105
Diabetes	84 (17.5%)	29 (17.1%)	55 (17.8%)	0.838
Previous myocardial infarction	79 (16.5%)	37 (21.8%)	42 (13.6%)	0.021
Previous CABG	17 (3.5%)	10 (5.9%)	7 (2.3%)	0.041
Previous PCI	94 (19.6%)	42 (24.7%)	52 (16.8%)	0.038
Treatment with aspirin	138 (28.8%)	61 (35.9%)	77 (24.9%)	0.011
Primary PCI < 24 h	432 (90.2%)	161 (94.7%)	271 (87.7%)	0.014
Surgery	10 (2.1%)	1 (0.6%)	9 (2.9%)	0.106
Time to presentation > 24 h	80 (16.7%)	23 (13.5%)	57 (18.4%)	0.167
Transfer from other hospitals	81 (16.9%)	25 (14.7%)	56 (18.1%)	0.340
Intra-hospital deaths	38 (7.9%)	25 (14.7%)	56 (18.1%)	0.218
Killip class > 2	48 (10.0%)	14 (8.2%)	34 (11%)	0.334
Intensive coronary unit stay length, days	3 (2–4)	4 (3–5)	3 (2–4)	<0.001
Continuous veno-venous hemofiltration	11 (2.3%)	3 (1.8%)	8 (2.6%)	0.754
**Hemodynamic support**				0.369
No support	406 (84.8%)	149 (87.6%)	257 (83.2%)	
IABP	68 (14.2%)	19 (11.2%)	49 (15.9%)	
ECMO	5 (1.0%)	2 (1.2%)	3 (1%)	
Mechanical ventilation	61 (12.7%)	19 (11.2%)	42 (13.6%)	0.448
Ventricular arrhythmias	55 (11.5%)	17 (10.0%)	38 (12.3%)	0.450
Atrial fibrillation	77 (16.1%)	27 (15.9%)	50 (16.2%)	0.932
Pulmonary edema	64 (13.4%)	21 (12.4%)	43 (13.9%)	0.630
AV block/PM	30 (6.3%)	13 (7.6%)	17 (5.5%)	0.354
Number of disease vessels				0.433
1	202 (42.2%)	65 (38.2%)	137 (44.3%)	
2	158 (33.0%)	60 (35.3%)	98 (31.7%)	
3	119 (24.8%)	45 (26.5%)	74 (23.9%)	
TIMI flow-grade pre-PCI				0.228
0	337 (70.3%)	111 (65.3%)	226 (73.1%)	
1	19 (4.0%)	6 (3.5%)	13 (4.2%)	
2	65 (13.6%)	29 (17.1%)	36 (11.7%)	
3	58 (12.1%)	24 (14.1%)	34 (11.0%)	
TIMI flow-grade post-PCI				0.011
0	4 (0.8%)	1 (0.6%)	3 (1.0%)	1.000
1	4 (0.8%)	3 (1.8%)	1 (0.3%)	0.130
2	22 (4.6%)	14 (8.2%)	8 (2.6%)	0.005
3	449 (93.7%)	152 (89.4%)	297 (96.1%)	0.004
Inotropes	68 (14.2%)	20 (11.8%)	48 (15.5%)	0.258
eGFR < 60 at presentation	71.4 ± 22.3	70.9 ± 21.1	71.7 ± 23.0	0.734
BNP at presentation	222 [69–452]	270 [100–843]	206 [62–421]	0.037
LVEF at presentation	45.3 ± 11.7	46.6 ± 11.6	44.6 ± 11.7	0.168
Wall motion score index at presentation	1.68 ± 0.44	1.63 ± 0.42	1.70 ± 0.44	0.116
LVEF at stabilization	50.1 ± 11.1	51.1 ± 11.5	49.4 ± 10.9	0.169
Wall motion score index at stabilization	1.60 ± 0.48	1.60 ± 0.49	1.60 ± 0.48	0.986
Pericardial effusion	48 (10.0%)	17 (10.0%)	31 (10.0%)	1.000
Intraventricular thrombosis	21 (4.4%)	2 (1.2%)	19 (6.1%)	0.010
Mechanical complications	15 (3.1%)	3 (1.8%)	12 (3.9%)	0.276
SARS-CoV-2 infection	7 (1.5%)	/	7 (2.3%)	/

Values are mean ± SD, n (%), or median (25th–75th percentile). CABG, coronary artery bypass graft; PCI, percutaneous coronary intervention; TIMI, Thrombolysis In Myocardial Infarction; LVEF, left ventricle ejection fraction; IABP, intra-aortic balloon pump; ECMO, extracorporeal membrane oxygenation; AV block, high grade atrio-ventricular block; PM, pacemaker; BNP, B-type natriuretic peptide; GFR, glomerular filtration rate.

Regarding the second wave of the COVID-19 pandemic in Italy, from October 1, 2020, to February 28, 2021 (Figure 1), in which there was a huge SARS-CoV-2 diffusion, 142 STEMI patients were admitted to our center. During the second wave of the pandemic, two STEMI patients (1.7%) had a concomitant infection with SARS-CoV-2. Compared to the same non-COVID-19 temporal window (from October 1, 2019, to February 28, 2020), patients admitted during the second wave presented more frequently with symptoms of onset-to-door time longer than 24 h (26.1 vs. 10.3%, *p* = 0.009). The rate of reperfusion by primary PCI within 24 h of the onset of symptoms was lower during the second wave of the pandemic than in the same pre-pandemic time window (85.2 vs. 97.1%, *p* = 0.009). Inotropic drugs were significantly more frequent during the second wave than during the pre-pandemic period (19.0 vs. 7.4%, *p* = 0.028). Pericardial effusion and LV thrombosis were higher in STEMI patients admitted during the second-wave period than in those admitted before COVID-19 (14.1 vs. 7.4%, *p* = 0.159; 7.7% vs. 1.5%, *p* = 0.108, respectively), although these differences were not significant (Table 2).

**TABLE 2:** Patient’s baseline and clinical characteristics before the pandemic and during the second COVID-19 wave.

	All patient (*n* = 210)	Before pandemic (*n* = 68)	During second wave (*n* = 142)	*P*-value
Demographic characteristics				
Age, years	67 ± 12	67 ± 13	67 ± 12	0.804
Female	51 (24.3%)	17 (25.0%)	34 (23.9%)	0.867
Body mass index, kg/m^2^	26.4 ± 4.0	26.4 ± 4.1	26.5 ± 3.9	0.860
Cardiovascular risk factors				
Hypertension	121 (57.6%)	40 (58.8%)	81 (57.0%)	0.807
Current smoking	53 (25.2%)	18 (26.5%)	35 (24.6%)	0.950
Dyslipidaemia	83 (39.5%)	32 (47.1%)	51 (35.9%)	0.122
Diabetes	32 (15.2%)	10 (14.7%)	22 (15.5%)	0.882
Previous myocardial infarction	37 (17.6%)	18 (26.5%)	19 (13.4%)	0.020
Previous CABG	6 (2.9%)	5 (7.4%)	1 (0.7%)	0.014
Previous PCI	43 (20.5%)	20 (29.4%)	23 (16.2%)	0.026
Treatment with aspirin	57 (27.1%)	28 (41.2%)	29 (20.4%)	0.002
Primary PCI < 24h	187 (89.0%)	66 (97.1%)	121 (85.2%)	0.009
Surgery	6 (2.9%)	0 (0.0%)	6 (4.2%)	0.180
Time to presentation > 24h	44 (21.0%)	7 (10.3%)	37 (26.1%)	0.009
Transfer from other hospitals	32 (15.2%)	9 (13.2%)	23 (16.2%)	0.576
Intra-hospital deaths	17 (8.1%)	2 (2.9%)	15 (10.6%)	0.058
Killip class > 2	23 (11.0%)	4 (5.9%)	19 (13.4%)	0.164
Intensive coronary unit stay length, days	3 (2–4)	4 (3–4)	3 (2–4)	0.002
Continuous venovenous hemofiltration	6 (2.9%)	1 (1.5%)	5 (3.5%)	0.660
Hemodynamic support				0.295
No support	177 (84.3%)	61 (89.7%)	116 (81.7%)	
IABP	32 (15.2%)	7 (10.3%)	25 (17.6%)	
ECMO	1 (0.5%)	0 (0.0%)	1 (0.7%)	
Mechanical ventilation	22 (10.5%)	5 (7.4%)	17 (12.0%)	0.306
Ventricular arrhythmias	20 (9.5%)	4 (5.9%)	16 (11.3%)	0.315
Atrial fibrillation	39 (18.6%)	12 (17.9%)	27 (19.0%)	0.812
Pulmonary edema	25 (11.9%)	7 (10.3%)	18 (12.7%)	0.618
AV block/PM	12 (5.7%)	7 (10.3%)	5 (3.5%)	0.048
Number of disease vessels				0.436
1				
2				
3				
TIMI flow-grade pre-PCI				0.228
0	136 (64.8%)	39 (57.4%)	97 (68.3%)	
1	10 (4.8%)	5 (7.4%)	5 (3.5%)	
2	33 (15.7%)	13 (19.1%)	20 (14.1%)	
3	31 (14.8%)	11 (16.2%)	20 (14.1%)	
TIMI flow-grade post-PCI				0.044
0	1 (0.5%)	0 (0.0%)	1 (0.7%)	1.000
1	2 (1%)	1 (1.5%)	1 (0.7%)	0.544
2	6 (2.9%)	5 (7.4%)	1 (0.7%)	0.014
3	201 (95.7%)	62 (91.2%)	139 (97.9%)	0.061
Inotropes	32 (15.2%)	5 (7.4%)	27 (19.0%)	0.028
eGFR < 60 at presentation	70.9 ± 22.8	69.2 ± 22.0	71.7 ± 23.2	0.467
BNP at presentation	159 [47–372]	375 [102–942]	135 [41–332]	0.013
LVEF at presentation	45.5 ± 10.2	46.8 ± 9.2	44.8 ± 10.6	0.215
Wall motion score index at presentation	1.69 ± 0.41	1.59 ± 0.36	1.73 ± 0.43	0.036
LVEF at stabilization	49.9 ± 11.2	52.1 ± 12.0	48.8 ± 10.7	0.090
Wall motion score index at stabilization	1.61 ± 0.49	1.57 ± 0.50	1.63 ± 0.48	0.536
Pericardial effusion	25 (11.9%)	5 (7.4%)	20 (14.1%)	0.159
Intraventricular thrombosis	12 (5.7%)	1 (1.5%)	11 (7.7%)	0.108
Mechanical complications	10 (4.8%)	0 (0.0%)	10 (7.0%)	0.032
SARS-CoV-2 infection	2 (1.0%)	/	2 (1.4%)	/

Values are mean ± SD, n (%), or median (25th–75th percentile). CABG, coronary artery bypass graft; PCI, percutaneous coronary intervention; TIMI, Thrombolysis In Myocardial Infarction; LVEF, left ventricle ejection fraction; IABP, intra-aortic balloon pump; ECMO, extracorporeal membrane oxygenation; AV block, high grade atrio-ventricular block; PM, pacemaker; BNP, B-type natriuretic peptide; and GFR, glomerular filtration rate.

Moreover, MC occurred more often in patients admitted during the second wave of the COVID-19 pandemic than in those admitted before the pandemic (7.0 vs. 0.0%, *p* = 0.032) (Table 2). Finally, in-hospital deaths (10.6 vs. 2.9%, *p* = 0.058) tended to be higher during the second wave than in the pre-pandemic period (Table 2). Additionally, the wall motion score index at presentation was significantly higher in STEMI patients admitted during the second wave of the pandemic than before the pandemic period (1.73 ± 0.43 vs. 1.59 ± 0.36, *p* = 0.036) (Table 2).

**FIGURE 3:**
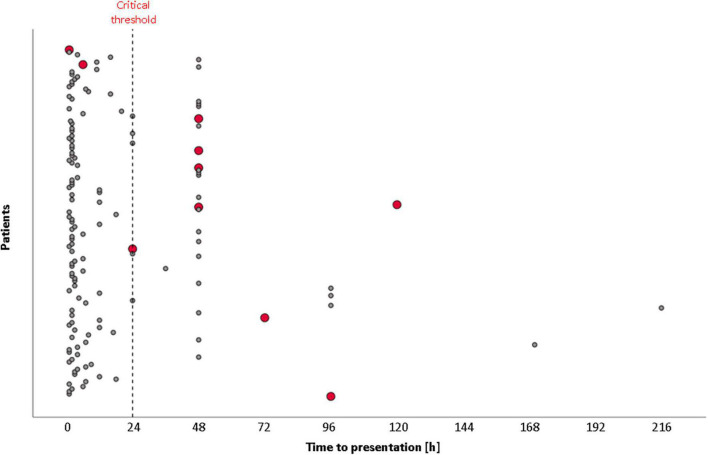
Time from symptoms’ onset to hospital presentation in STEMI patients during the second wave of COVID-19. Red points represent STEMI patients with mechanical complications.

Looking at the 10 STEMI patients with MC during the second wave of the pandemic, we observed that acute free wall rupture, ventricular septal rupture, and papillary muscle rupture occurred in two (1.4%), four (2.8%), and four (2.8%) patients, respectively (Supplementary Table 1). Seven cases underwent surgery; three survived, while three others were not operated on and died. The baseline characteristics of patients with MC are reported in Table 3. Overall, 50% were men, and the mean age was 77 ± 5 years; 40.0% had pericardial effusion, 30.0% had an apical aneurism, 20.0% reported an apical thrombosis, 70.0% died during the hospital stay, and 80.0% had a symptom onset to hospital arrival time ≥ 24 h (Figure 3). Due to late arrival and/or no indications for other clinical and angiographic reasons, eight out of these ten cases did not undergo PCI. None were confirmed positive for SARS-CoV-2 infection.

**TABLE 3:** Patient characteristics of STEMI patients with mechanical complications admitted during the second COVID-19 wave.

	During the second wave (*n* = 10)
Age, years	77 ± 5
Female	5 (50.0%)
Time to presentation	
>24 h	8 (80.0%)
>48 h	7 (70.0%)
>72 h	3 (30.0%)
Killip class > 2	7 (70.0%)
Infarct location	
Anterior	2 (20.0%)
Anterolateral	1 (10.0%)
Posterior/inferior-lateral	2 (20.0%)
Posterior/inferior	5 (50.0%)
TIMI flow-grade pre-PCI	
0	10 (100%)
1	0 (0%)
2	0 (0%)
3	0 (0%)
TIMI flow-grade post-PCI	
0	0 (0%)
1	1 (10.0%)
2	0 (0%)
3	9 (90%)
Intensive coronary unit stay length, days	3 [1–5]
Intra-hospital deaths	7 (70.0%)
Hemodynamic support in shock (IABP, ECMO)	9 (90.0%)
Previous myocardial infarction	0 (0.0%)
Previous CABG	0 (0.0%)
Previous PCI	0 (0.0%)
SARS-CoV-2 infection	0 (0.0%)
Pericardial effusion	4 (40.0%)
Apical aneurism	3 (30.0%)
Apical thrombosis	2 (20.0%)

Values are mean ± SD, n (%), or median (25th–75th percentile). TIMI, Thrombolysis In Myocardial Infarction; IABP, intra-aortic balloon pump; ECMO, extracorporeal membrane oxygenation; PCI, percutaneous coronary intervention; CABG, coronary artery bypass graft.

In an analysis limited to STEMI patients admitted during the second wave of the pandemic, including all variables that clustered patients with MCs with a *p* < 0.05, the multivariable analysis demonstrated that transfer from other hospitals (OR: 5.53 [95% CI: 1.03–29.77]) and cardiogenic shock (OR: 18.92 [95% CI: 1.68–212.56]) were independent predictors of MCs (Table 4).

**TABLE 4:** Univariable and multivariable predictors of risk of mechanical complications were observed during the second COVID-19 wave.

	Univariable analysis		Multivariable analysis	
		
	OR (95% CI)	*P*-value	OR (95% CI)	*P*-value
Age, years	1.100 (1.027–1.178)	0.006		
Female	3.552 (0.962–13.114)	0.057		
Primary PCI < 24 h	0.085 (0.022–0.338)	< 0.001		
Time to presentation > 24h	10.500 (2.530–43.570)	0.001		
Transfer from other hospitals	16.917 (3.968–72.117)	< 0.001	5.531 (1.028–29.768)	0.046
Killip class > 2	23.333 (5.328–102.191)	< 0.001		
Hemodynamic support in shock (IABP, ECMO)	60.882 (7.251–211.206)	< 0.001	18.920 (1.684–212.561)	0.017
eGFR < 60 at presentation	6.725 (1.646–27.482)	0.008		
BNP at presentation	1.002 (1.001–1.004)	0.003		
Pericardial effusion	4.833 (1.230–18.999)	0.024		

PCI, percutaneous coronary intervention; IABP, intra-aortic balloon pump; ECMO, extracorporeal membrane oxygenation; BNP, B-type natriuretic peptide; GFR, glomerular filtration rate.

## Discussion

This study aimed to investigate the impact of the COVID-19 pandemic on AMI patients, specifically on clinical and echocardiographic complications, including MCs rate, admitted to a macro-hub center during the COVID-19 pandemic, with a special focus on the second wave of COVID-19.

Indeed, the Lombardy Region government built up a big hub-and-spoke model to converge treatment of ACS in 13 dedicated centers active 24/7 in the region, implementing the availability of intensive care unit beds in general hospitals converted to COVID-19 treatment. Our institute was one of the four selected macro-hub centers in Milan (Lombardy, Italy) for cardiological or cardiac surgical emergencies (four in the entire Lombardy Region). Due to this specific hub-and-spoke organization, we admitted and treated more AMI patients during the COVID-19 pandemic, compared to the decrease in PCI procedures and AMI admissions in our region and other countries. Therefore, we had the unique opportunity to investigate the time trends of admissions for AMI, rate of major complications, and in-hospital mortality, as well as detailed clinical and echocardiographic data before and during the pandemic and according to the first or second COVID-19 wave. By focusing our analysis on the second wave of the pandemic, we observed that the main finding of this study is the clear demonstration that the incidence of MC significantly increased with the delay between symptoms onset and hospital admission. In particular, 10 AMI patients had an MC in the second wave (7%) vs. 0 in the pre-pandemic matched period. Of the 10 cases with MC (Figure 3), 1 had a delay of >24 h and 7 > 48 h. In-hospital mortality in the second wave of the COVID-19 period was 10.6 vs. 2.9% of the pre-pandemic period, and a time to presentation > 24 h was observed in 26.1 vs. 10.3% of the second-wave and pre-pandemic periods, respectively, thereby markedly reducing the rate of PCI < 24 h.

All clinical and echocardiographic findings showed a trend toward higher severity of the myocardial injury and related hemodynamic consequences in patients admitted during the COVID-19 pandemic and, in particular, in those admitted during the second wave, as reported in Table 2. Indeed, Killip class, B-type natriuretic peptide at presentation, inotropes therapy, LV wall motion score, pericardial effusion, and LV thrombus formation confirmed the late arrival to the hospital and no/or late reperfusion dramatically affected hemodynamic status and significantly increased the rates of major AMI complications.

Mechanical complications in the PCI era has a low incidence (0.3–1%) (7, 11). Bouisset et al. (12) recently showed an increased MC in STEMI patients admitted during the pandemic, related to pre-hospital delay in the last 6 months in France (1.3%). The delay between symptoms onset and first medical contact was significantly longer among patients with MC: 801 [210–3406] vs. 135 [60–369] min. Similar to our data, they also observed that LV aneurysm and LV thrombus were more frequent among patients with MC: 4.8 vs. 0.4% and 3.6 vs. 1.3%, respectively. The main difference between that study and ours is that it was a national cohort (65 centers), and half of their cohort was recruited during the first wave of the COVID-19 pandemic in France. Moreover, that period was associated with a strong decrease in hospitalizations in France for ACS. As in our data, very few cases had COVID-19 infection. Thus, MC seems not to be directly related to virus infection and inflammation.

Previous reports described an increasing trend in the cases of MC during the COVID-19 pandemic compared to a parallel time frame before the pandemic. Bryndza et al. (13) observed a decrease among patients presenting with STEMI during the case period (18.6%), with a trend toward a more frequent incidence of MC, correlated to a longer delay between symptoms onset and hospital admission. Compared with the corresponding period before pandemic, Kitahara et al. (14) detected an increase in STEMI late admission during the COVID-19 outbreak (25.4 vs. 14.2%). Patient delay translated into a higher proportion of patients presenting MC upon hospital arrival (14.3 vs. 3.6%). Similarly, Lin et al. (15) showed that although there was no reduction in AMI hospital admissions during the COVID-19 pandemic, a longer onset-to-hospital time may increase MC incidence.

Our data reinforce the well-known relationship between myocardial damage and late or lack of myocardial reperfusion. Studies in the pre-thrombolytic era showed rates of MC as high as 6% with transmural MI, while the incidence in the reperfusion PCI era was as low as < 1%. Elbadawi et al. (7), in a 13-year observational analysis of about 9 million hospitalizations with AMI, showed that the rate of MC was 0.27% in the STEMI cohort and 0.06% in the NSTEMI cohort, with no significant changes over time. The overall in-hospital mortality rate among patients who developed MCs was 42.4% among patients with STEMI and 18.0% among those with NSTEMI, with no changes over time. In our study, the finding of 7% of MC is similar to the pre-PCI era. Moreover, in-hospital mortality (10.6%) was similar to pre-PCI (9–10% with fibrinolytic therapy and 11.5% among patients without therapy). Interestingly, our institute’s in-hospital mortality before the pandemic was 2.9%.

Damluji et al. (16), in a scientific statement from the American Heart Association on MC of Acute Myocardial Infarction, reported that advances in primary prevention resulted in a significant decline in the age- and sex-adjusted incidence of AMI during the past decades. However, despite such improvements, large infarcts, late hospital presentation, and a lack of tissue-level reperfusion attributable to no-reflow or poor coronary flow after PCI remain risk factors for MC, hemodynamic instability, and pump failure. The mortality rate for the 3 MC was very high, even in the PCI era. Although a modern pharmacological and non-pharmacological approach to cardiogenic shock includes papillary muscle rupture (10–40%), ventricular septal defect (30–40%), and rupture of the free LV wall (50%). These data refer to the “ideal” medical and surgical approach in the pre-COVID-19 period with early recognition due to diagnosis, multidisciplinary stakeholder involvement in medical resuscitation, stabilization, and patient-centered planning and timing of appropriate surgical intervention, percutaneous technologies, and mechanical circulatory support. Despite having all these potentialities and approaches in our institute during the second pandemic period, the overall mortality in MC was very high, thus further underlining the severity of the clinical and hemodynamic status in our cases and the differences related to very late arrival at the hospital.

### Hub-and-spoke model and impact on MI treatment

Italy was the first western country to be hit by the pandemic. From February 2020, the COVID-19 infection rate rose significantly within a few weeks, prompting a lockdown from March to May (69 consecutive days overall). Therefore, a regional law redesigned the hub-and-spoke system for time-dependent diseases to better allocate resources for COVID-19 patients. The model adopted by Lombardy, the most densely populated region in Italy (approximately 10 million inhabitants with 20 different cardiac surgical units and 55 catheterization laboratories, of which most perform 24/7 PCI), was to concentrate cardiac emergencies in four vascular surgery hubs.

Lombardy was the first European region to reorganize its cardiovascular emergency system in reaction to the spread of the COVID-19 infection. This well-defined pathway for cardiovascular emergencies was efficient in allowing surgical and percutaneous procedures in cases with ACS and rapidly reacting to the first wave of the pandemic. Similarly, when the second wave occurred, the same model was maintained, and our study documented that we admitted 309 patients with AMI in 4 months. Despite this organization, as already demonstrated in the first wave (17), the hub-and-spoke model should be associated with a strong media campaign on the importance of early hospital admission for suspected ACS in a COVID-19-free hospital environment. However, independent of patients’ consideration of the hospital as a possible contagion area, which triggered many patients’ fear of going to the emergency rooms, our data also showed the critical role of transfer from other hospitals. On multivariate analysis, this variable impacted MC, which should stimulate a more rapid transfer to hub hospitals based on symptoms and/or triage, including ECG in the organization of transport in emergency suspected MI.

Several countries organized specific systems to react to this emergency (18–20). As a concern in our region, Bonalumi et al. (21) reported that the proposed hub-and-spoke organization system efficiently safeguarded access to heart and vascular surgery.

In conclusion, despite an efficient hub-and-spoke model for cardiovascular emergencies in our region (Lombardy, Italy), even in the second wave of the COVID-19 pandemic, persistent late admission to the hospital resulted in high STEMI mortality and a high incidence of MC. In parallel with reorganization and optimal resource allocation in a pandemic, health authorities should provide effective programs to increase awareness of the symptoms and timely and standard-of-care treatment of AMI to the general public.

## Data availability statement

The raw data supporting the conclusions of this article will be made available by the authors, without undue reservation.

## Ethics statement

The studies involving human participants were reviewed and approved by Centro Cardiologico Monzino IRCCS. The patients/participants provided their written informed consent to participate in this study.

## Author contributions

MarP, AF, NC, GT, FC, PR, ST, GM, and MauP contributed to the conception and design of the study. AF, NC, MI, PR, ST, and GM organized the database. MarP performed the statistical analysis. MarP and MauP wrote the first draft of the manuscript. GT and AF wrote sections of the manuscript. All authors contributed to the manuscript revision, read, and approved the submitted version.
