# Supplementary material for: Outcomes and mechanical complications of acute myocardial infarction during the second wave pandemic in a Milan HUB center for cardiac emergencies
Source: Front Cardiovasc Med. 2022 Oct 3;9:950952. doi: 10.3389/fcvm.2022.950952 (PMC9573996; doi:10.3389/fcvm.2022.950952)
Supplement: Supplementary file 1 [file Table_1.pdf]

Outcomes and Mechanical Complications of Acute Myocardial Infarction during the Second Wave Pandemic in a Milan HUB Center for Cardiac Emergencies

Supplemental appendix

Supplemental Table 1. Characteristics of STEMI patients with mechanical complications during the second wave of the COVID-19 pandemic.

Age	Sex	Date of admission	Mechanical complication	Angiography	PCI	TIMI	Culprit	Surgery	Death
61	F	March 2019	PMR	✓	✓	3	RCA		✓
67	M	May 2019	FWR	✓	✓	2	RCA		✓
65	F	June 2019	VSD	✓	✓	2	LAD		✓
56	M	June 2020	PMR	✓		/	RI		
81	F	June 2020	VSD	✓		/	no significant stenosis	✓	✓
70	M	September 2020	VSD		✓	/	RCA		✓
86	F	November 2020	PMR			/	NA		✓
74	M	Decembre 2020	VSD	✓		/	LAD	✓	✓
78	F	December 2020	FWR			/	/	✓	
82	M	Decembre 2020	VSD	✓		/	PDA	✓	✓
83	F	January 2021	PMR			1	RCA	✓	
70	F	January 2021	PMR	✓	✓	1	CFX	✓	
80	M	January 2021	PMR	✓		1	M0	✓	✓
74	M	February 2021	VSD			/	RCA	✓	✓
77	F	February 2021	FWR			/	/		✓

PCI = percutaneous coronary intervention; TIMI = Thrombolysis In Myocardial Infarction; F, female; M, male; VSD = ventricular septal defect; PMR = papillary muscle rupture; FWR= free wall rupture; RCA right coronary artery; LAD=left anterior descendent; RI= ramus intermediate; PDA posterior descending artery; CFX, circumflex artery
